# Proposing a Compartmental Model for Leprosy and Parameterizing Using Regional Incidence in Brazil

**DOI:** 10.1371/journal.pntd.0004925

**Published:** 2016-08-17

**Authors:** Rebecca Lee Smith

**Affiliations:** Department of Pathobiology, University of Illinois College of Veterinary Medicine, Urbana, Illinois, United States of America; Fondation Raoul Follereau, FRANCE

## Abstract

Hansen’s disease (HD), or leprosy, is still considered a public health risk in much of Brazil. Understanding the dynamics of the infection at a regional level can aid in identification of targets to improve control. A compartmental continuous-time model for leprosy dynamics was designed based on understanding of the biology of the infection. The transmission coefficients for the model and the rate of detection were fit for each region using Approximate Bayesian Computation applied to paucibacillary and multibacillary incidence data over the period of 2000 to 2010, and model fit was validated on incidence data from 2011 to 2012. Regional variation was noted in detection rate, with cases in the Midwest estimated to be infectious for 10 years prior to detection compared to 5 years for most other regions. Posterior predictions for the model estimated that elimination of leprosy as a public health risk would require, on average, 44–45 years in the three regions with the highest prevalence. The model is easily adaptable to other settings, and can be studied to determine the efficacy of improved case finding on leprosy control.

## Author Summary

Control of Hansen’s disease, or leprosy, requires understanding how quickly the infection moves through the population and how long it takes to detect the disease. These rates vary regionally, resulting in differences in the number of people detected with disease each year. We have estimated the risk of infection and the rate of detection for this disease in each of the 5 regions of Brazil. This allowed us to predict the long-term impact of Brazil’s current leprosy control program, which found that some regions of Brazil will require 44–45 years to eliminate leprosy as a public health risk, primarily due to the long delay in detection of cases.

## Introduction

Hansen’s disease (HD, or leprosy) is a chronic progressive disease caused in Brazil by infection with *Mycobacterium leprae*. Transmission is most likely through nasal droplets [1], and is associated with socioeconomic status [2]. While leprosy is curable through chemotherapy [3], detection is often delayed [1], leading to more serious sequelae (including disfigurement and disability). The World Health Organization (WHO) has set a goal for elimination of leprosy as a public health problem, defined as a prevalence of <1/10,000 [4]. Several countries, including Brazil, have failed to meet that goal [5].

The Brazilian leprosy control program has been successful in decreasing the incidence of leprosy, but the prevalence remains high in 2 regions [5]. Movement towards elimination seems to have stagnated in these regions, possibly due to a downgrading of the importance of case finding [6]. Treatment of leprosy has been decentralized, so regional differences in case detection, and disease progression are to be expected [7]. Infection hotspots have also been noted in Brazil [8], leading to regional and sub-regional differences in transmission rates [5]. These may be related to socioeconomic factors, as a systematic review has found that socioeconomic inequalities associated with leprosy were large [9]. Prediction models must take these regional differences into account in order to accurately represent these differences and identify possible control points.

A number of models of leprosy have been proposed [10–21], and 6 of the base models from these studies were recently fitted to regional data from Brazil [22]. However, only one model takes into account much of the recent research on leprosy susceptibility [10,18], and it is an agent-based model that relies on specific population structures; the results of this model are quite useful on a regional level [23,24], but have not been applied to national-level results. The goal of this research is to produce a compartmental model that represents current understanding of leprosy susceptibility and pathogenesis, but that is also easily adaptable to different populations. Unknown parameters for this model will be fitted to regional incidence data from Brazil and analyzed to determine differences in control efficacy and their effect on the elimination target and long-term control.

## Methods

All human data was anonymized at the source before usage [25]. As these data were publicly available and fully anonymized, no institutional review board approval was required.

**Fig 1:**
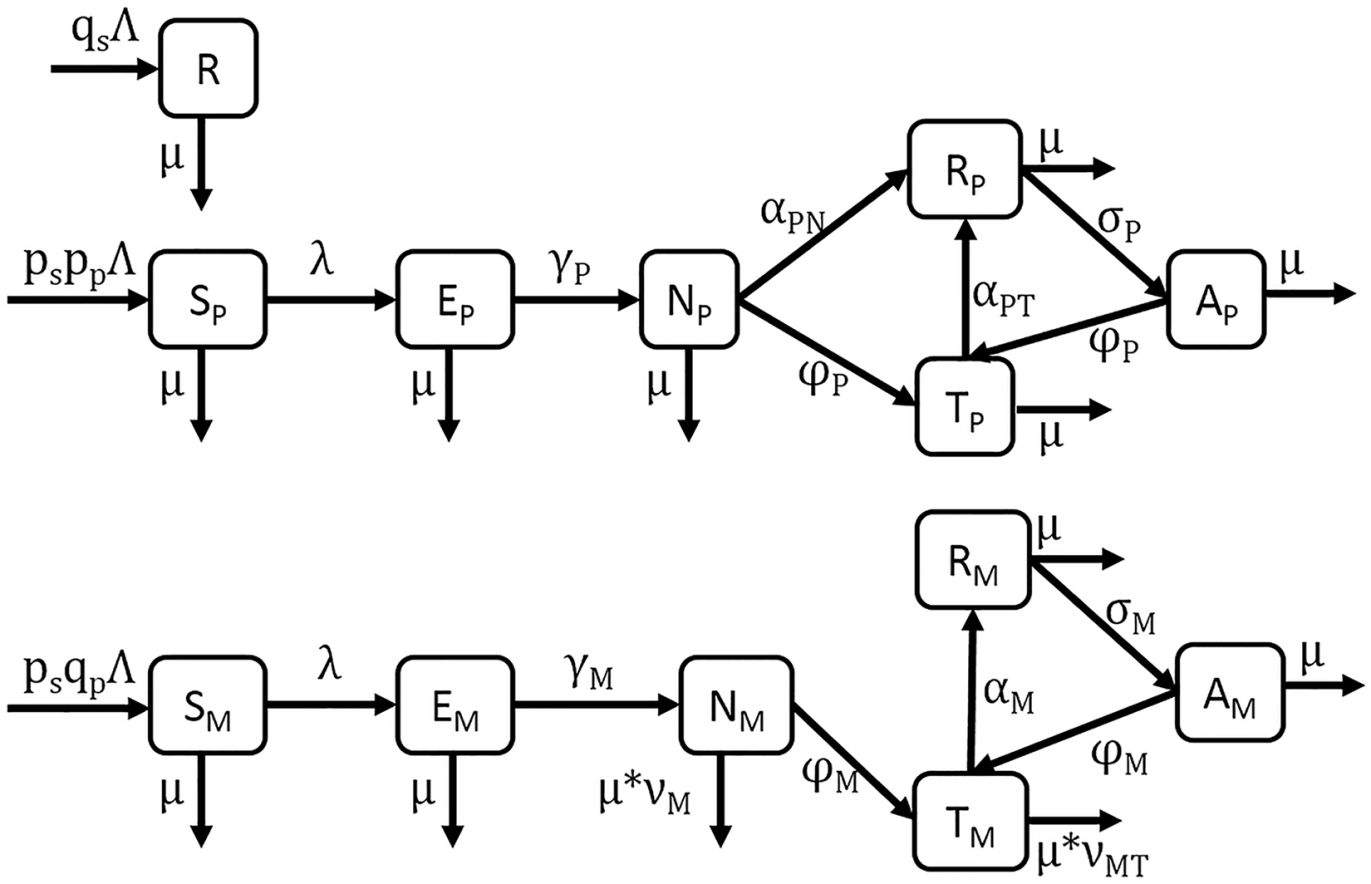
A compartment model of Hansen’s disease.

A deterministic compartment model of leprosy (Fig 1) was designed to take into account current understanding of the disease. Briefly, individuals are divided into 3 categories: resistant (*R*, with probability *q*_*S*_ *= 1-p*_*s*_), susceptible to paucibacillary infection (S_P_, with probability *p*_s_*p_*p*_), and susceptible to multibacillary infection (S_M_, with probability *p*_s_*q_*p*_ where *q*_*P*_ *= 1-p*_*p*_). Resistance, *q*_*s*_, is meant to convey both genetic resistance and socioeconomic protective factors [13,18]. Resistance to multibacillary infection, *p*_*p*_, is meant to convey genetic resistance [18]; this value is higher than the observed proportion of new cases that are PB (0.8 vs. 0.54), but the discrepancy is explained by the high rate of self-cure among PB cases (*α*_*PN*_). Resistant individuals (*R*) enter and leave the population without infection. Individuals with susceptibility to leprosy but genetic resistance to MB disease enter the paucibacillary (PB) track as susceptible (*S*_*P*_). They may be exposed (*E*_*P*_) at rate *λ* and eventually develop symptomatic PB disease (*N*_*P*_) at rate *γ*_*P*_. Paucibacillary disease either self-heals at rate *α*_*PN*_ or is detected and leads to treatment (*T*_*P*_) at rate *φ*_*P*_, either of which results in recovery (*R*_*P*_) at rate *α*_*PT*_. Recovered individuals may relapse to refractory disease (*A*_*P*_) at rate *σ*_*P*_, from which they can be detected and return to treatment at rate *φ*_*P*_. Individuals with a genetic susceptibility to MB disease enter the population as susceptible (*S*_*M*_) and may become exposed (*E*_*M*_) at rate *λ*. Exposed individuals develop multibacillary disease (*N*_*M*_) at rate *γ*_*M*_ and are diagnosed and entered into treatment (*T*_*M*_) at rate *φ*_*M*_. Treated individuals recover or leave treatment (*R*_*M*_) at rate *α*_*M*_ and may relapse to refractory disease (*A*_*M*_) at rate *σ*_*M*_, from which they may be detected and return to treatment at rate *φ*_*M*_. Multibacillary individuals are subject to a death rate that is proportionately higher than the general population, at *ν*_*M*_ for untreated individuals and *ν*_*MT*_ for treated individuals.

The force of infection, *λ*, is calculated as

$$\lambda =\frac{\beta_{P}\left(N_{P}+\theta_{1}T_{P}+A_{P}\right)+\beta_{M}\left(N_{M}+\theta_{2}T_{M}+A_{M}\right)}{N}$$ (1)

for the density-dependent model, where *N* is the total population size, *β*_*P*_ is the contribution of PB individuals to the force of infection, *β*_*M*_ is the contribution of MB individuals to the force of infection, *θ*_*1*_ and *θ*_*2*_ are the proportional decreases in infectiousness due to treatment of PB and MB individuals, respectively. Total population size (*N*) is calculated as the sum of all the compartments, and varies over time as births and deaths occur. For the sake of simplification, it is assumed that treated individuals are quickly rendered non-infectious [3] and therefore that *θ*_*1*_ = *θ*_*2*_ = 0. Thus, the force of infection becomes

$$\lambda =\frac{\beta_{P}\left(N_{P}+A_{P}\right)+\beta_{M}\left(N_{M}+A_{M}\right)}{N}$$ (2)

in the density-dependent model. Good estimates were available in the literature for most system parameters (see Table 1). However, estimates were unavailable for the transmission coefficients (*β*_*P*_ and *β*_*M*_) and the true case detection rates (*φ*_*P*_ and *φ*_*M*_), as these are likely to vary by locality and can be difficult to measure directly. These parameters were therefore estimated using the Sequential Monte Carlo approximate Bayesian Computation (SMC ABC) algorithm [26,27] applied to Brazilian incidence data, as described previously [22]. Briefly, the annual incidence of PB leprosy, $inc_{obs}^{PB}\left(y\right)$, and the annual incidence of MB leprosy,$inc_{obs}^{MB}\left(y\right)$, were obtained for each of the 5 regions of Brazil between 2000 and 2012 [28]. For each region, an initial parameter set (n = 100,000) was sampled from the prior distributions of the estimated parameters (a uniform distribution with a non-informative range, Table 1), and in subsequent SMC particles (rounds), parameter sets were sampled from the immediately previous particle with a perturbation kernel. Each parameter set was used to simulate the incidence values between 2000 and 2010 (allowing 2011–2012 to be used for unconstrained validation), where incidence was assumed to be new individuals entering the treated category (*T*_*P*_ or *T*_*M*_) from the untreated category (*N*_*P*_ or *N*_*M*_). A distance function was calculated using the equation

$$d=\sum_{y=2000}^{2010}{\left(inc_{obs}^{PB}\left(y\right)-inc_{pred}^{PB}\left(y\right)\right)}^{2}+{\left(inc_{obs}^{MB}\left(y\right)-inc_{pred}^{MB}\left(y\right)\right)}^{2}$$ (3)

where $inc_{pred}^{PB}\left(y\right)$ was assumed to be equal to *φ*_*P*_*N*_*P*_*(y)*, or the number of new PB cases entering treatment in year *y*, and $inc_{pred}^{MB}\left(y\right)$ was assumed to be equal to *φ*_*M*_*N*_*M*_*(y)*, or the number of new MB cases entering treatment in year *y*. It was assumed that recurrent infections (from *A*_*P*_ or *A*_*M*_) were not included in the observed incidence. A parameter set was accepted if *d<τ*, where *τ* was set equal to the 75^th^ percentile of *d* in the previous particle. In each particle, the algorithm was repeated until 100,000 parameter sets were accepted; 10 particles were produced, with the 10^th^ particle used to form the posterior distribution. The perturbation kernel was set to be a uniform distribution with a range limited by ± the variance of each parameter in the previous particle.

**Table 1:** Starting parameter values and ranges for a compartmental model of Hansen’s Disease. Regional values are set based on the demographics of the area providing observed data for fitting. Fitted values (unfitted assumption in parentheses) are estimated using Approximate Bayesian Computation.

Symbol	Description	Value
Λ	Rate at which individuals enter the population (year^-1^) [28]	Regional
μ	Mortality rate (year^-1^) [28]	Regional
β_P_	Effective contact rate for PB (year^-1^) [29]	Fitted (0.3, range 1e-5:5)
**$\beta_{P}^{f}$**	Effective contact rate for PB in a frequency-dependent model (year^-1^)	Fitted (7.5e-9, range 1.25e-13:6.25e-8)
β_M_	Effective contact rate for MB (year^-1^) [29]	Fitted (0.15, range 1e-5:5)
**$\beta_{M}^{f}$**	Effective contact rate for MB in a frequency-dependent model (year^-1^)	Fitted (3.75e-9, range 1.25e-13:6.25e-8)
φ_M_	Case finding rate for MB (year^-1^) [11]	Fitted (0.5, range 0.2–4)
φ_P_	Case finding rate for PB (year^-1^) [11]	Fitted (0.5, range 0.2–4)
p_s_	Probability that an individual is susceptible to infection [11]	0.1
γ_M_	Rate of progression to MB (year^-1^) [11]	0.1
γ_P_	Rate of progression to PB (year^-1^) [11]	0.28
p_p_	Probability that an individual is susceptible to PB infection only [11]	0.8
θ_1_	Reduction factor of β for treated over untreated PB [30]	0
θ_2_	Reduction factor of β for treated over untreated MB [30]	0
α_M_	Recovery rate from treated MB (year^-1^) [1]	1
α_PN_	Self-recovery rate from PB (year^-1^) [11]	0.224
α_PT_	Recovery rate from treated PB (year^-1^) [1]	2
σ_M_	Rate of relapse to MB after recovery (year^-1^) [11]	0.009
σ_P_	Rate of relapse to PB after recovery (year^-1^) [11]	0.001
v_M_	Disease-induced proportional increase in mortality rate in untreated MB (year^-1^) [31]	3.5
ν_MT_	Disease-induced proportional increase in mortality rate in treated MB (year^-1^) (assumed)	1

PB = paucibacillary disease

MB = multibacillary disease

Initial values in each of the compartments were determined analytically based on the parameters and observed prevalence. As the duration of treatment in MB disease is twice the duration of treatment in PB disease under MDT, the observed prevalence was assumed to be divided between *T*_*M*_*(0)* and *T*_*P*_*(0)* with the ratio $2*\frac{incidence\left(MB\right)}{incidence\left(PB\right)}$. The ratio of *N*_*i*_*(0)*:*T*_*i*_*(0)*, *E*_*i*_*(0)*:*N*_*i*_*(0)*, *R*_*i*_*(0)*:*T*_*i*_*(0)*, and *A*_*M*_*(0)*:*N*_*M*_*(0)*, where *i* ∈ {M,P}, were set empirically in a multi-step process similar to that described previously [22]. Briefly, the ratios were adjusted manually for each region such that the model, simulated with the assumed values in Table 1, predicted the incidence of both PB and MB cases in that region with less than 10% deviation from the observed values in 2000 (the first year of observation) and 2002 (the year of peak incidence in most regions). The model was then fitted with the initial population distribution determined by these ratios, and the median of the estimated distribution for each fitted parameter was used to predict incidence of both PB and MB cases in each region. If the predicted incidences in 2000 or 2002 deviated from observed values in any region by more than 10%, the ratios were again adjusted manually to correct the deviation and the model was re-fitted. This process repeated until the median of the estimated distribution for each fitted parameter was able to predict incidence of PB and MB cases in each region with less than 10% deviation from observed values in both 2000 and 2002.

As density-dependent transmission was assumed, but is known to be a simplification of true human contact rates [32], the above process was repeated for a frequency-dependent transmission model. In this model, the force of infection *λ*_*f*_ becomes

$$\lambda_{f}=\beta_{P}^{f}\left(N_{P}+A_{P}\right)+\beta_{M}^{f}\left(N_{M}+A_{M}\right)$$ (4)

where $\beta_{P}^{f}$ and $\beta_{M}^{f}$ are adjusted from the density-dependent model to account for population size. The results of the frequency and density dependent models were compared using Bayes factor analysis, in which the Bayes factor was the ratio of the summed distance in all regions, corrected for differences in regional population size, across a weighted sample of 1,000 posterior parameter sets.

The results of the best-fitting regional model (frequency or density dependent) were examined for similarity between distributions, and 3 hierarchical fittings were considered: transmission parameters (*β*_*M*_, *β*_*P*_) shared across regions (V1), transition parameters (*φ*_*M*_, *φ*_*P*_) shared across regions (V2), all parameters (*β*_*M*_, *β*_*P*_, *φ*_*M*_, *φ*_*P*_) shared across regions (V3), and sharing no parameters (the regional model described above, V4). In the hierarchical models, the distance function was altered to

$$d=\sum_{r}\sum_{y=2000}^{2010}{\left(inc_{obs}^{PB}\left(y,r\right)-inc_{pred}^{PB}\left(y,r\right)\right)}^{2}+{\left(inc_{obs}^{MB}\left(y,r\right)-inc_{pred}^{MB}\left(y,r\right)\right)}^{2}/N_{r}$$ (5)

where r represents the region and *N*_*r*_ is the population of region *r* in 2000. Hierarchical models were compared to each other and the regional model using Bayes factor analysis, in which the Bayes factor was the ratio of the summed distance in all regions, corrected for differences in regional population size, across a sample of 1,000 posterior parameter sets weighted by the inverse of their summed regional distances (Eq 5).

In order to check the consistency of the model results, data were simulated for each region using the median of the best fitted value from the preferred hierarchical model. These data were then used to repeat the full model selection and parameterization process, including hierarchical model selection and parameterization. Results were compared to the simulated input values.

Posterior predictions were produced using a weighted sample of 1,000 parameter sets from the posterior distribution of both the preferred hierarchical model and the regional model, and outcomes of interest were predicted from this sample. Outcomes were the time to elimination in years (t_elim_) and the predicted incidence overall and of MB and PB cases in the year 2050 (i_2050_, iM_2050_, and iP_2050_, respectively).

All models and fitting were performed in R 3.0.3,[33] which was accessed through the Revolution R Analytics interface (copyright 2014 Revolution Analytics, Inc.).

## Results

The median and range of each parameter for the each of the model fits are shown in Table 2. Transmission and transition parameters were similar between the density-dependent and frequency-dependent models. Bayes factor analysis identified the frequency-dependent model as having the lowest summed deviance from the observed incidence. As a result, the frequency-dependent model was used for hierarchical model fitting.

**Table 2:** Posterior distribution median and 95% prediction intervals determined by ABC fitting of Approximate Bayesian Computation models for Hansen’s Disease to data from the 5 regions of Brazil. Version 4 consisted of fitting the regional best-fit model to each region’s observed data separately with both frequency and density-dependent transmission assumptions; all other versions used a hierarchical structure with density-dependent transmission in which at least some parameters were shared across regions, and fitting was done simultaneously across all 5 regions. Mean error refers to the average value of d per iteration of each version, based on a sample of 1,000 iterations, with confidence intervals based on 100 samples of 100 iterations each.

V^a^	Reg.^c^	β_M_	β_P_	φ_M_	φ_P_	Mean Error
**1**	**N**			0.41 (0.1–2.6)	0.45 (0.11–2.9)	
	**NE**			0.01 (0.01–0.01)	0.01 (0.01–0.012)	2.1e-14
	**SE**	1.4 (0.011–2.5)	0.75 (0.0012–2.4)	0.01 (0.01–2.7)	0.01 (0.01–2.7)	2e-14-
	**S**			0.01 (0.01–2.7)	0.01 (0.01–2.6)	-2.3e-14)
	**MW**			0.47 (0.11–3)	0.41 (0.057–2.7)	
**2**	**N**	1.5 (0.0031–2.5)	1.5 (0.004–2.5)			
	**NE**	0.84 (0.0031–2.4)	1.3 (0.0013–2.5)			1.3e-14
	**SE**	1.1 (0.0024–2.5)	1.2 (0.0015–2.5)	0.19 (0.15–0.25)	0.23 (0.17–0.32)	(1.2e-14-
	**S**	1.1 (0.004–2.5)	1.2 (0.0073–2.5)			1.3e-14)
	**MW**	1.5 (0.0057–2.5)	1.3 (0.0085–2.5)			
**3**	**All**	1.6 (0.0036–2.5)	1 (0.0036–2.5)	0.19 (0.14–0.28)	0.23 (0.16–0.35)	1.7e-14 (1.7e-14-1.8e-14)
**4_f_**^b^	**N**	2.2 (1.4–2.5)	1.4 (0.0027–2.4)	0.2 (0.2–0.22)	0.52 (0.39–0.73)	
	**NE**	2.1 (1.5–2.5)	1.1 (0.59–1.6)	0.2 (0.2–0.21)	0.2 (0.2–0.2)	1.0e-14
	**SE**	1.5 (0.68–2.5)	0.66 (0.007–1.1)	0.51 (0.44–0.63)	0.2 (0.2–0.21)	(1.0e-14-
	**S**	1.8 (0.76–2.5)	0.86 (0.0024–1.7)	0.46 (0.43–0.52)	0.2 (0.2–0.2)	1.0e-14)
	**MW**	1.7 (1.2–2.5)	0.26 (9.1e-3-0.56)	0.01 (0.01–0.01)	0.01 (0.01–0.01)	
**4_d_**	**N**	0.7 (0.5–0.8)	0.7 (8.9e-4-0.8)	0.2 (0.2–0.22)	0.52 (0.39–0.73)	
	**NE**	2.5 (1.8–3.0)	1.4 (0.7–1.9)	0.2 (0.2–0.21)	0.2 (0.2–0.2)	1.4e-14
	**SE**	2.7 (1.2–4.6)	1.2 (1.3e-2-2.1)	0.51 (0.44–0.63)	0.2 (0.2–0.21)	(1.4e-14-
	**S**	1.1 (0.5–1.6)	0.5 (1.6e-3-1.1)	0.46 (0.43–0.52)	0.2 (0.2–0.2)	1.4e-14)
	**MW**	0.1 (8.2e-3-0.2)	0.01 (4.8e-5-0.1)	0.01 (0.01–0.01)	0.01 (0.01–0.01)	

^a^Version of the hierarchical structure sharing parameters across 5 regions of Brazil: 1) all parameters shared; 2) transmission parameters shared; 3) transition parameters shared; 4_f_) no parameters shared, frequency-dependent transmission; 4_d_) no parameters shared, density-dependent transmission

^b^Density-dependent transmission parameters have been transformed to be comparable to frequency-dependent transmission parameters by multiplying the estimated values by the population size in the year 2000.

^c^Region: N = North (Acre, Amapá, Amazonas, Pará, Rondônia, Roraima, and Tocantins States), NE = Northeast (Alagoas, Bahia, Ceará, Maranhão, Paraíba, Pernambuco, Piauí, Rio Grande do Norte, and Sergipe states), SE = Southeast (Espírito Santo, Minas Gerais, Rio de Janeiro, and São Paulo states), S = South (Paraná, Rio Grande do Sul, and Santa Catarina states), MW = Midwest (Goiás, Mato Grosso, Mato Grosso do Sul, and Distrito Federal states)

Bayes factor analysis identified the regional version as having the lowest summed deviance from the observed incidence, although the preference for the regional version was not strong (Bayes Factor of 1.2 to 2.1, compared to the hierarchical models). Transmission coefficients were estimated to be similar in all regions even in the regional model, but transition rates had high variability between regions. Transition rates were lower in the Midwest for all individuals, higher in the South and Southeast for MB individuals, and higher in the North for PB individuals.

**Fig 2:**
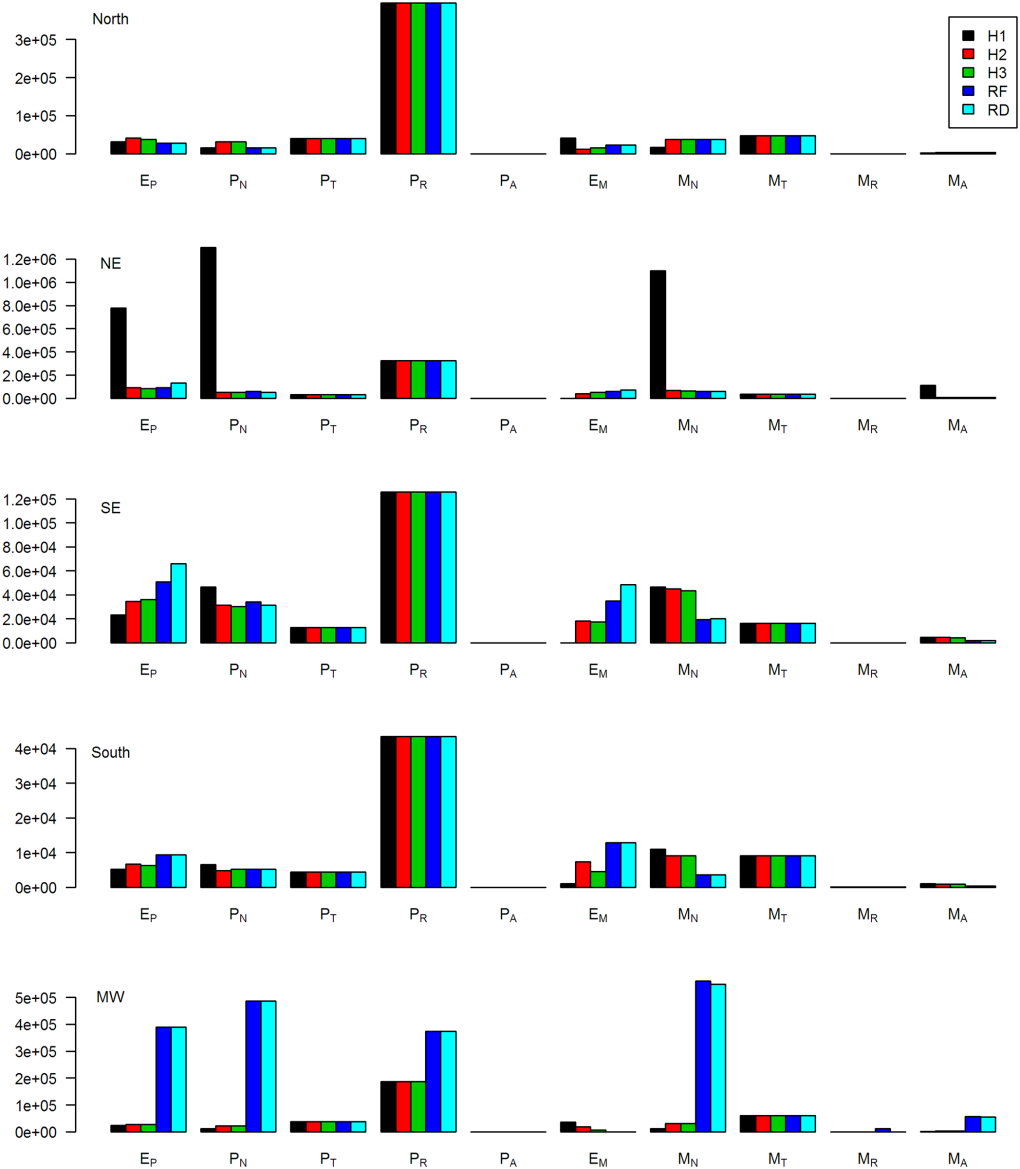
Number of initial population in infected categories for a dynamic model of *Mycobacterium leprae* in Brazilian regions. The colors represent different model fits (black H1: fitted transmission parameters shared across all regions, red H2: fitted transition parameters shared across all regions, green H3: all fitted parameters shared across all regions, dark blue RF: no fitted parameters shared across regions, and light blue RD: no fitted parameters shared across regions and density-dependent transmission). Each row is a different region. The infected categories are: E_P_, latent paucibacillary (PB); P_N_, undetected PB; P_T_, treated PB; P_R_, recovered PB; P_A_, recurrent PB; E_M_, latent multibacillary (MB); M_N_, undetected MB; M_T_, treated MB; M_R_, recovered MB; and M_A_, recurrent MB.

The final distribution of the initial population, as determined by the empirical process, is shown in Fig 2 for each region and model fit. The number of latent and undetected individuals was the most variable across models, with the density-dependent model requiring higher numbers of latent individuals to reproduce the initial and peak incidence in each region.

**Fig 3:**
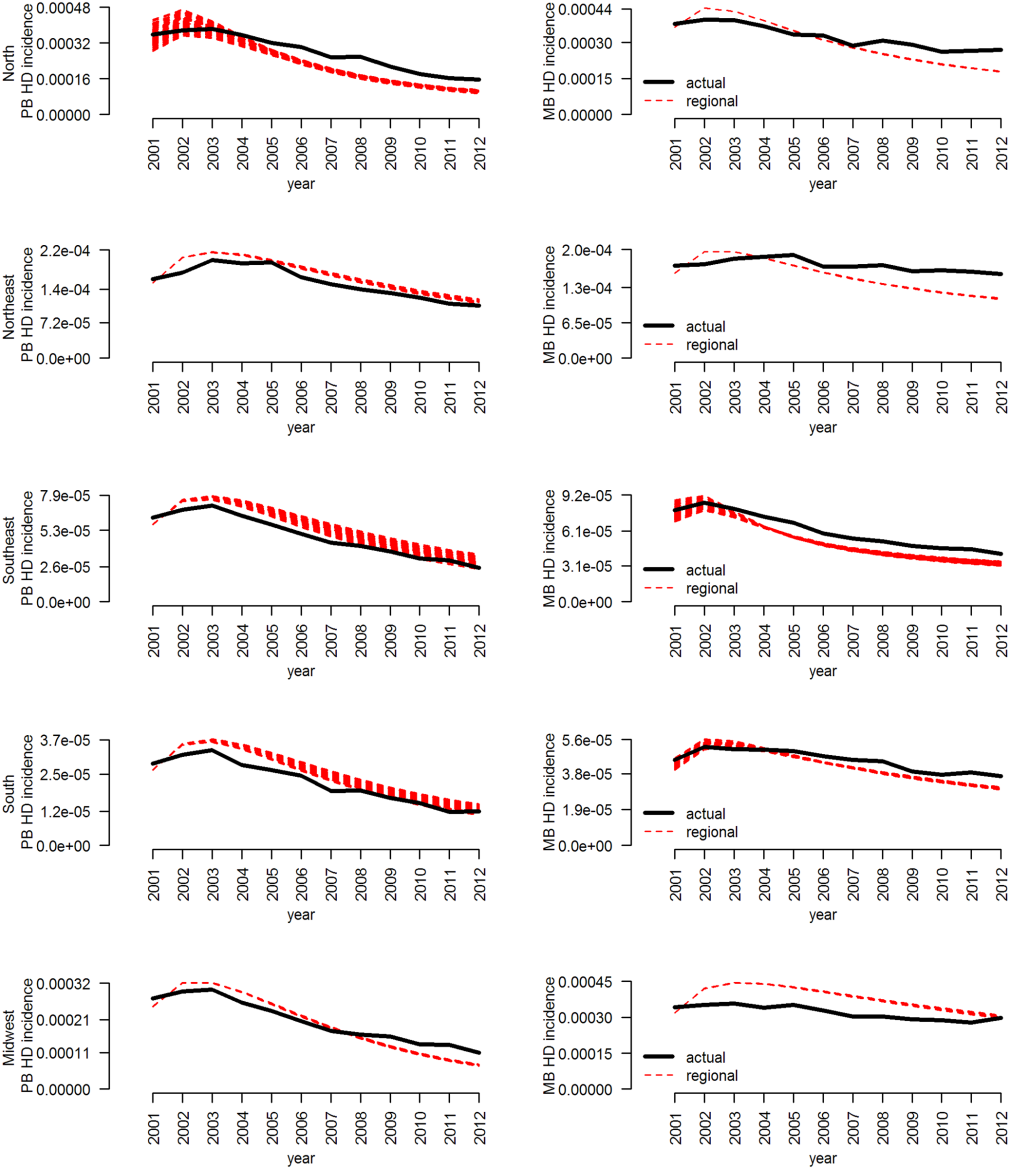
Posterior predictions for incidence and multibacillary (MB) incidence of the compartmental model of Hansen’s disease for the regions of Brazil, compared to the observed values for 2000–2012. Unknown parameters were fitted to each region individually. All models were fit using Approximate Bayesian Computation with the Sequential Monte Carlo algorithm.

Posterior predictions for the preferred hierarchical model and the regional model are shown in Fig 3 and Table 3. The results show that the fit underestimated PB incidence in the North and Midwest and MB incidence in most regions in later years. The South and Southeast reported incidences below the elimination threshold in 2001, and this was also predicted to be possible by the model, although the average time to elimination was predicted to be 2002 and 2007, respectively. On average, the North and Midwest were predicted to reach the elimination threshold by 2045, while the Northeast was predicted to reach the elimination threshold by 2044. However, the ranges of values were wide, indicating that the Northeast could require up to the year 2053 to reach the elimination threshold.

**Table 3:** Posterior predictions (mean and range) from the regional model for Hansen’s Disease fit to regional data from Brazil. Incidence of Hansen’s Disease in the year 2050 is reported overall (i_2050_) and for multibacillary (iM_2050_) and paucibacillary (iP_2050_). The time to elimination (t_elim_) was calculated as the year in which overall incidence was ≤1/10,000, starting from 2001.

Region	i_2050_ (/10,000)	iM_2050_ (/10,000)	iP_2050_ (/10,000)	t_elim_
**North**	0.91 (0.83–0.98)	0.57 (0.55–0.59)	0.34 (0.29–0.4)	45 (40–49)
**Northeast**	0.99 (0.92–1.1)	0.54 (0.52–0.56)	0.45 (0.41–0.5)	44 (38–53)
**Southeast**	0.22 (0.17–0.28)	0.14 (0.12–0.17)	0.071 (0.042–0.11)	7.3 (7–8)
**South**	0.16 (0.15–0.17)	0.13 (0.12–0.13)	0.031 (0.024–0.039)	1 (1–1)
**Midwest**	0.89 (0.88–0.89)	0.66 (0.63–0.69)	0.22 (0.2–0.25)	45 (44–46)

Simulation results (Table 4) show that the model was able to predict the simulated values in most cases. The exceptions were the values of φ_M_ and φ_P_, which tended to overestimate the true value, and the values of β_M_ and β_P_ in the density-dependent model, which tended to underestimate the true value.

**Table 4:** Posterior distribution median and 95% prediction intervals determined by ABC fitting of Approximate Bayesian Computation models for Hansen’s Disease to data simulated by the best-fit model. The values fit were β_M_ = 1.9, β_P_ = 1.2, φ_M_ = 0.2, and φ_P_ = 0.2 for all but the North, where φ_2_ = 0.46. Version 4 consisted of fitting the regional best-fit model to each region’s observed data separately with both frequency and density-dependent transmission assumptions; all other versions used a hierarchical structure with density-dependent transmission in which at least some parameters were shared across regions, and fitting was done simultaneously across all 5 regions. Values in bold italics contained the simulated value within their range. Mean error refers to the average value of d per iteration of each version, based on a sample of 1,000 iterations, with confidence intervals based on 100 samples of 100 iterations each.

V^a^	Region	β_M_	β_P_	φ_M_	φ_P_	Mean Error
**1**	**North**			***0*.*44 (0*.*23–1*.*2)***	***0*.*42 (0*.*2–1*.*2)***	
	**Northeast**			***0*.*2 (0*.*2–2*.*1)***	***0*.*2 (0*.*2–0*.*86)***	5.7e-15
	**Southeast**	***1*.*2***	***0*.*89***	***0*.*2 (0*.*2–2*.*8)***	***0*.*2 (0*.*2–2*.*7)***	(5.4e-15-
	**South**	***(0*.*00049–2*.*5)***	***(0*.*00049–2*.*4)***	***0*.*2 (0*.*2–2*.*6)***	***0*.*2 (0*.*2–2*.*6)***	6.1e-15)
	**Midwest**			0.45 (0.22–1.3)	***0*.*2 (0*.*2–0*.*33)***	
**2**	**North**	***1*.*6 (0*.*00073–2*.*5)***	***1*.*3 (0*.*005–2*.*5)***			
	**Northeast**	***1*.*1 (0*.*00073–2*.*5)***	***1*.*6 (0*.*00022–2*.*5)***			3.7e-15
	**Southeast**	***1*.*3 (0*.*0038–2*.*5)***	***1*.*2 (0*.*0031–2*.*5)***	0.5 (0.39–0.69)	0.47 (0.35–0.65)	(3.5e-15-
	**South**	***1*.*3 (0*.*0054–2*.*5)***	***1*.*2 (0*.*00095–2*.*5)***			3.8e-15)
	**Midwest**	***1*.*5 (2*.*8e-05-2*.*5)***	***1*.*5 (0*.*00043–2*.*5)***			
**3**	**All**	***2*.*1 (0*.*24–2*.*5)***	***1*.*6 (3e-04-2*.*5)***	0.49 (0.39–0.7)	0.46 (0.34–0.66)	3.6e-15 (3.3e-15-3.7e-15)
**4**_**f**_^b^	**North**	***2 (0*.*0086–2*.*5)***	***1*.*2 (0*.*00018–2*.*5)***	0.5 (0.4–0.67)	0.49 (0.39–0.64)	
	**Northeast**	***2*.*4 (1*.*8–2*.*5)***	2.1 (1.3–2.4)	0.48 (0.4–0.64)	0.44 (0.36–0.56)	3e-15
	**Southeast**	***2*.*4 (1*.*8–2*.*5)***	2.2 (1.4–2.5)	0.49 (0.36–0.83)	0.27 (0.2–0.38)	(2.8e-15-
	**South**	***2*.*1 (0*.*2–2*.*5)***	***1*.*3 (0*.*00069–2*.*5)***	0.49 (0.31–0.97)	0.46 (0.22–1.2)	3.1e-15)
	**Midwest**	***2 (0*.*00078–2*.*5)***	***1*.*2 (0*.*00024–2*.*4)***	0.5 (0.4–0.7)	0.49 (0.37–0.69)	
**4**_**d**_	**North**	***1*.*4 (0*.*13–3)***	***0*.*85 (1e-5-2*.*3)***	0.48 (0.39–0.62)	***0*.*44 (0*.*37–0*.*63)***	
	**Northeast**	1e-5 (1e-5-1.5)	1e-5 (1e-5-0.6)	***0*.*2 (0*.*2–0*.*2)***	***0*.*2 (0*.*2–0*.*2)***	1.7e-14
	**Southeast**	***2*.*8 (2–4*.*7)***	***1*.*3 (1e-5-2*.*4)***	***0*.*2 (0*.*2–0*.*2)***	***0*.*2 (0*.*2–0*.*2)***	(1.6e-14-
	**South**	0.36 (1e-5-1.5)	1e-5 (1e-5-0.97)	***0*.*2 (0*.*2–0*.*2)***	***0*.*2 (0*.*2–0*.*21)***	1.7e-14)
	**Midwest**	1e-5 (1e-5-1e-5)	1e-5 (1e-5-1e-5)	***0*.*2 (0*.*2–0*.*2)***	***0*.*2 (0*.*2–0*.*2)***	

^a^Version of the hierarchical structure sharing parameters across 5 regions of Brazil: 1) all parameters shared; 2) transmission parameters shared; 3) transition parameters shared; 4_f_) no parameters shared, frequency-dependent transmission; 4_d_) no parameters shared, density-dependent transmission

^b^Frequency-dependent transmission parameters have been transformed to be comparable to density-dependent transmission parameters.

## Discussion

This study presents a compartmental model for Hansen’s Disease that takes into account the current understanding of the disease but that is computationally simple and easy to adapt. The structure of this model differs from that of Meima et al. [11] in 2 ways. First, this model assumes that the 90% of people who never develop leprosy are inherently resistant, rather than self-healing. Second, this model assumes that the 80% of susceptibles who will only develop PB disease are again inherently resistant to MB disease. That allows us to separate the relapsed cases appropriately, such that only those recovered from MB disease relapse to MB disease. These assumptions also allow for including differences of susceptibility in a compartmental model, which is less computationally intensive, easier to fit to data, and easy to adapt to other populations or across larger and more diverse regions. However, Ridley-Jopling classification allows for borderline cases which can cross between PB and MB groups during relapse.[34] While it would be advantageous to capture the full diversity of leprosy presentation in future models, the parameterization of such models will require improved reporting; at present, Brazil reports only the PB and MB classifications of detected cases.[25]

Many studies at the national or regional level will report a “case detection rate”. This, however, is not the true case finding rate represented by φ_M_ and φ_P_ in this model. It is, instead, the annual observed incidence, and uses the population as the denominator, not the delay in diagnosis. This is an important difference: a high “case detection rate” could indicate an outbreak rather than fast detection, while the true case finding rate only represents the time necessary to identify a clinical case. We found that the range of potential case finding rates was fairly similar across most regions, but that cases were estimated to be infectious for 5 years in most cases before treatment was initiated. This agrees with the findings of multiple studies [35,36], where people delayed seeking treatment partly from an assumption that the symptoms were not serious and potentially from a worry of the stigma attached to a diagnosis [37]. It also falls into the range assumed by previous models within Brazil for this time period [24]. In a modeling study, Fischer et al. [10] found that contact tracing was important to avoid the diagnostic delay; contact tracing was decreased in India in order to meet WHO case detection rate goals, and the result was treatment delays [38], which would produce a long-term effect of higher incidence and, eventually, higher case detection rates. Importantly, our model predicted that cases in the Midwest were infectious for an average of 100 years before detection, an unrealistic value indicating that the true detection rate of cases is too low to estimate properly. If true, this could indicate a public health failing that should be addressed. This difference in detection rate between regions could be socio-economic in origin, as the three regions predicted to have low detection rates (Northeast, North, and Midwest) also have lower GDP per capita. Similar regional health disparities have been noted for ischaemic heart disease [39] and laryngeal cancer [40] mortality, suggesting regional disparities in health care [41].

The decision was made to compare density- and frequency-dependent models, despite the fact that leprosy is considered to be a disease of close contact and therefore would be classically considered to have density-dependent transmission. This is due to the limitations of a model such as this, caused by the homogeneous mixing assumption, in capturing the limited number of close contacts any individual is likely to have. Thus, while a disease may be truthfully density-dependent, it may behave mathematically as a frequency-dependent disease. The results of this study show that the density-dependent model was slightly preferred to the frequency-dependent model. This question would not arise with an individual-based model, such as SIMCOLEP [42], but those models are not as easy to adapt as they must rely on population-specific characteristics in their design, which may require parameters that are not locally available. The goal of this study was to provide an adaptable model that was still able to capture the regional dynamics of leprosy spread. The preference of the regional model supported this decision, but the preference was not strong, indicating that some national-level models may be as informative as the regional models.

Several models assume that PB individuals are non-infectious [11]. We observed that PB individuals did contribute to the force of infection, although with roughly half to two-thirds the strength of MB individuals. In other mycobacterial diseases, less infectious individuals have been found to be potentially important in maintaining the endemicity of the infection [43,44]. This highlights the importance of diagnosing and treating all cases; although the PB cases do not have as serious sequelae as the MB cases, they may serve to maintain the infection in a region. With regards to the intra-regional variation in transmission parameters, we found that higher transmission parameters were predicted in regions with higher incidence. This is to be expected, and highlights the ability of the model fitting to identify regional differences.

The results of the scenario analysis show that all regions are well on-track to eliminate leprosy, with the South and Southeast, which have the lowest incidence, likely to eliminate leprosy first. The posterior prediction plots (Fig 2) show that the fitted model estimated the observed decrease in PB incidence fairly accurately in most regions, but slightly overestimated the decrease in incidence of MB cases in all but the Midwest. It may be assumed, therefore, that these results are best-case scenarios for those regions. The predicted incidence in the Northern region is higher than has recently been predicted for Para State, which is within that region, but the time to the elimination target generally agrees between the two models [24]. All regions observed an increase in incidence up to 2003, followed by a slow decrease. This is likely due to the slow impact of control programs, rather than a change in case detection rates; chronic diseases with long latent periods, like leprosy, will require consistent control over long periods of time to reverse incidence trends.

It is important to note that the incidence of MB disease was predicted to be in the range of 13,983 to 14,913 new MB cases in Brazil in 2015. However, the North, Northeast, and Midwest are likely to require a much longer period to reach official elimination than the South and Southeast. The best way to decrease the time to elimination would be increasing the case finding rate [45]. This would also improve the level of disability in new cases, as delay in onset of treatment is a major cause of disability. Our results, therefore, indicate that the North, Northeast, and Midwest regions of Brazil would benefit from improving the true case finding rate, which we have estimated to be slow.
